# Catamenial pneumothorax with bubbling up on the diaphragmatic defects: a case report

**DOI:** 10.1186/s12905-021-01318-0

**Published:** 2021-04-20

**Authors:** Bo Dong, Chun-Li Wu, Yin-liang Sheng, Bin Wu, Guan-Chao Ye, Ya-Fei Liu, Shi-Hao Li, Lu Han, Yu Qi

**Affiliations:** Department of Thoracic Surgery, The First Affiliated Hospital of Zhengzhou University, No.1 Jianshe East Road, Zhengzhou, 450052 Henan Province China

**Keywords:** Case report, Catamenial pneumothorax, Endometriosis, Thoracic endometriosis syndrome TES

## Abstract

**Background:**

Catamenial pneumothorax is characterized by spontaneous recurring pneumothorax during menstruation, which is a common clinical manifestation of thoracic endometriosis syndrome. There are still controversies about its pathogenesis.

**Case presentation:**

A 43-year-old woman with a history of endometriosis came to our hospital due to recurring pneumothorax during menstruation. Uniportal Video-assisted Thoracoscopic Surgery (VATS) exploration was performed on the eve of menstruating. We thoroughly explored the diaphragm, visceral and parietal pleura: The lung surface was scattered with yellowish-brown implants; no bullae were found; multiple diaphragmatic defects were found on the dome. And surprisingly, we caught a fascinating phenomenon: Bubbles were slipping into pleural cavity through diaphragmatic defects. We excised the diaphragmatic lesions and wedge resected the right upper lung lesion; cleared the deposits and flushed the thoracic cavity with pure iodophor. Diaphragmatic lesions confirmed the presence of endometriosis, and interestingly enough, microscopically, endometrial cells were shedding with impending menses. After a series of intraoperative operations and postoperative endocrine therapy, the disease did not recur after a period of follow-up.

**Conclusion:**

We have witnessed the typical signs of catamenial pneumothorax at the accurate timing: Not only observed the process of gas migration macroscopically, but also obtained pathological evidence of diaphragmatic periodic perforation microscopically, which is especially precious and confirms the existing theory that retrograde menstruation leads to diaphragmatic endometriosis, and the diaphragmatic fenestration is obtained due to the periodic activities of ectopic endometrium.

## Background

Catamenial pneumothorax is a relatively rare type of Spontaneous Pneumothorax (SP), which was first reported by Maurer and his colleagues in 1958 [1]. Later, in 1972, Lillington combined their findings and named it [2]. The diagnosis of catamenial pneumothorax originally relied on clinical manifestations [3]: Women of childbearing age develop spontaneous recurrent pneumothorax within 24 h before or 72 h after the onset of menstruation. Later, when surgery was the preferred diagnostic and therapeutic measure of this recurrent disease, postoperative pathological and morphological changes gave us a clearer understanding.

The pathogenesis of catamenial pneumothorax is still unclear, the most accepted one is: Intra-abdominal endometriosis lesions retrograde to the diaphragm, resulting in acquired diaphragm perforation with the periodic activity of hormones. Perforated diaphragm or pathological evidence can support this inference.

## Case presentation

A 43-year-old woman with a history of endometriosis came to our hospital because of frequent chest stuffiness (attacking every three to six months), which can be traced back to 2 years ago. The X-ray demonstrated a right mild pneumothorax. Subsequently, this recurring symptom closely related to menstruation bothered her deeply. During this time, she tried closed drainage of pleural cavity, however, the frequent recurrence of her symptoms was bothersome. Chest CT scan showed a recurring pneumothorax on the right, no obvious bullae, we decided to explore the source of pneumothorax under uniportal VATS.

**Fig. 1:**
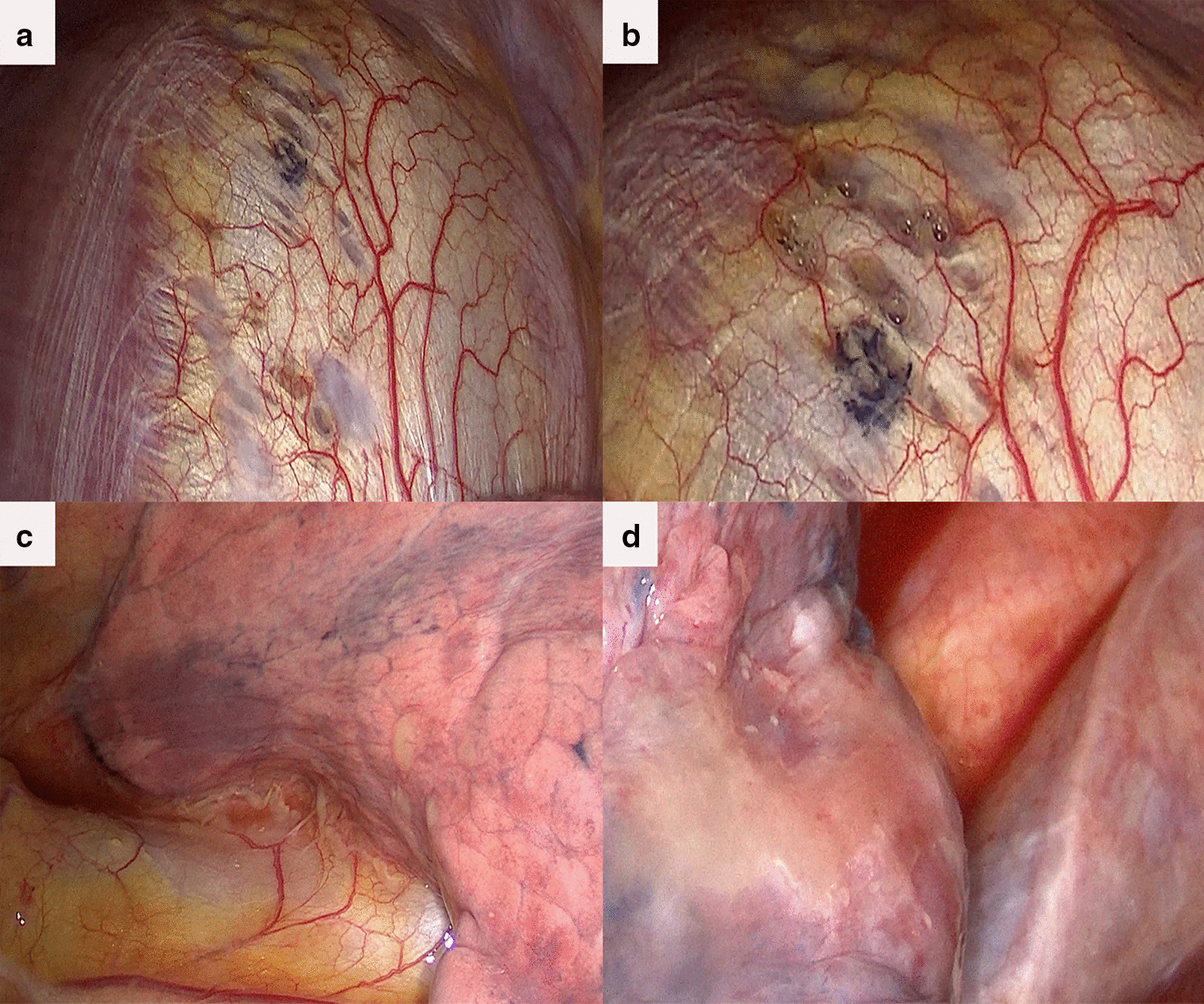
Intraoperative findings; **a** There were multiple defects in the diaphragmatic dome, and the swing of liver can be clearly seen along with breathing movement, **b** With breathing, bubbles were overflowing into the pleural cavity through the fenestrations, **c** Yellowish-brown nodules and fibrinous deposits scattering in the pleural cavity and on the visceral pleura. **d** Tight attachments of the upper right lung that cannot be washed away

We thoroughly explored the diaphragm, visceral and parietal pleura: no bullae were found; multiple diaphragmatic defects were found on the dome (Fig. 1a). And surprisingly, bubbles were slipping through the defects to the thoracic cavity (Fig. 1b); the visceral pleura was scattered with yellowish-brown implants (Fig. 1c). We excised the diaphragmatic lesions and wedge resected the right upper lung lesions (shown in Fig. 1d); cleared the deposits and rinsed pleural cavity with pure iodophor; wiped the parietal pleura with dry gauze to preclude hidden lesions.

**Fig. 2:**
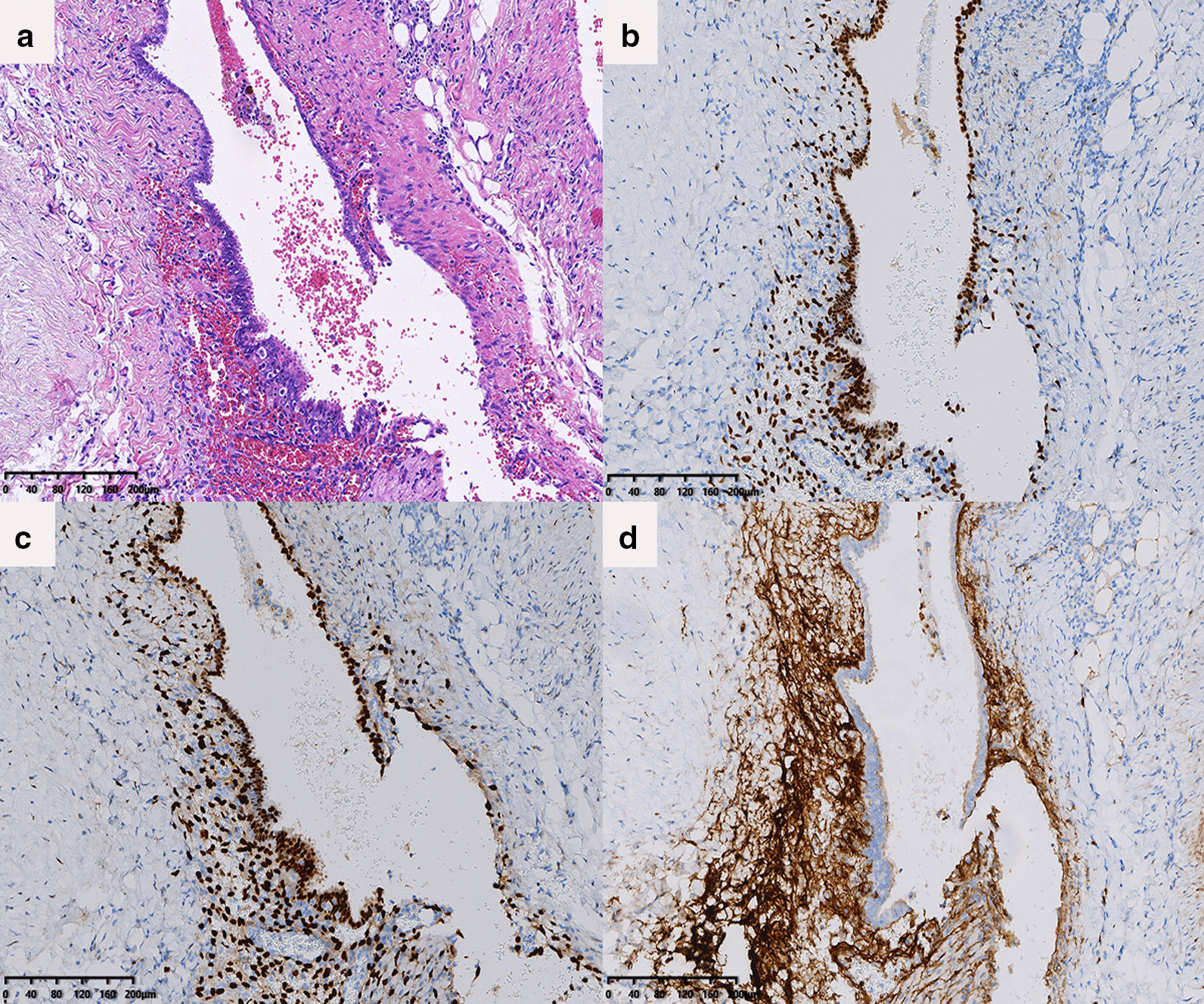
Pathological findings of diaphragmatic fenestrations; **a** Ectopic gland cells were presented, and sloughed cells could clearly be seen in the gland cavity (hematoxylin and eosin method). The gland cells were positive for **b** ER+, **c** PR+, **d** CD10+, further suggesting the presence of endometriosis

Histopathological examination revealed endometriosis in diaphragmatic lesions, periodic shedding of endometrial cells and a large number of blood cells can be seen in the gland cavity, immunohistochemistry showed CD10, ER and PR positive (Fig. 2), right upper lung lesions showed inflammation.

On the second day after operation, she had menstruation. Her postoperative process was uneventful. Subsequently, she received endocrine therapy (Gonadotropin Releasing Hormone agonist GnRH-a; 3.6 mg; once every 28 days, 4 times in total), after a period of follow-up, the pneumothorax did not recur.

## Discussion

Catamenial pneumothorax is characterized by spontaneous recurring pneumothorax during menstruation, which is a common clinical manifestation of thoracic endometriosis syndrome. Surgical treatment has become one of its standardized treatment methods, in previous literature reports, the diaphragmatic defect observed during surgery is the most common sign [4]. The most appropriate time for surgery is recommended during menstrual period, so that the typical signs and pathological findings can be observed more clearly. However, the testimony of gas migration is only an occasional capture in imaging [5]. In our case, we chose to operate on the eve of menstruation, the initial expectation was to confirm the existence of thoracic endometriosis via pathology. By chance, we witnessed the scene of bubbles boiling on the diaphragmatic defects and diaphragmatic deciduous ectopic gland cells under the microscope. Until now, there has not been such a typical discovery of this rare case.

The relationship between catamenial pneumothorax and endometriosis has been confirmed, in our case, the endometriosis history and pathological conclusions can also validate the speculation. Its pathogenesis can be explained by the “Determinant of uterineeutopic endometrium “as a clinical manifestation of TES: Eutopic endometrium is the root cause, its own characteristics determine the location of the thoracic lesions, diaphragm is the most common, and so do the lungs and the pleura. Meanwhile, about catamenial pneumothorax, there are mainly three related mechanisms [6]: 1. Anatomical theory: Retrograde menstruation resulting in subdiaphragmatic endometriosis, air migrates to pleural cavity through the genital tract and acquired diaphragmatic perforation during menstruation; 2. Metastasis theory: Ectopic endometrial tissue spreads to the thoracic cavity through blood or lymphatic vessels; 3. Physiological theory: The effects of prostaglandin F2.

During the operation, we found a large number of yellowish-brown nodules, considering that the visceral pleura was also involved. After repeatedly flushing the pleural cavity with sterile water, we noticed that there was yellowish-brown deposition tightly sticking to upper right lung, but the postoperative pathology revealed inflammatory exudation. We believe that the fibrin deposits and numerous yellow–brown nodules were exudate of the pleura and the previously shed endometrial tissue.

High recurrence rate is the main trouble in the treatment of catamenial pneumothorax. However, for patients with separate diaphragmatic lesion, there are reports in the previous literatures that surgical treatment usually has a very good prognosis [7, 8]. For this patient, in addition to removing the diaphragmatic lesions and reconstructing diaphragm, we also used pure iodophor flushing the thoracic cavity to promote pleural adhesions, which we believed that may be used as another preventive measure to form coagulated iodinated protein protective membrane on the lung surface. Carefully exploring the entire pleural cavity and wiping the parietal pleura with dry gauze to exclude other lesions. After the perioperative period, we added endocrine therapy. Through this series of systematic treatment measures, we believe that the efforts paid off.

Surgical exploration of catamenial pneumothorax is usually found with diaphragmatic fenestrations. However, in our case, the witness of bubbling up on the diaphragmatic defects and the positive pathological results have proved that we accurately grasped the timing of surgery. At this time, we discovered a phenomenon that has not been reported before and further clarified the pathogenesis of catamenial pneumothorax, which is still controversial until now.

## Data Availability

Data sharing is not applicable to this article as no datasets were generated or analysed during the current study.

## Abbreviations

VATS: Video-asisted thoracic surgery
SP: Spontaneous pneumothorax
CT: Computed tomography
TES: Thoracic endometriosis syndrome
GnRH-a: Gonadotropin releasing hormone agonist
